# Paternal perinatal stress is associated with children's emotional problems at 2 years

**DOI:** 10.1111/jcpp.13695

**Published:** 2022-10-10

**Authors:** Fiona L. Challacombe, Johanna T. Pietikäinen, Olli Kiviruusu, Outi Saarenpää‐Heikkilä, Tiina Paunio, E. Juulia Paavonen

**Affiliations:** ^1^ Section of Women's Mental Health IOPPN, King's College London London UK; ^2^ Department of Public Health and Welfare Finnish Institute for Health and Welfare Helsinki Finland; ^3^ Department of Psychiatry University of Helsinki and Helsinki University Central Hospital Helsinki Finland; ^4^ Pediatric Clinics Tampere University Hospital Tampere Finland; ^5^ Tampere Centre for Child Health Research University of Tampere and Tampere University Hospital Tampere Finland; ^6^ Department of Psychiatry and SleepWell Research Program, Faculty of Medicine University of Helsinki and Helsinki University Central Hospital Helsinki Finland; ^7^ Pediatric Research Center, Child Psychiatry University of Helsinki and Helsinki University Hospital Helsinki Finland

**Keywords:** Fathers, postpartum, stress, anxiety, infant, child development

## Abstract

**Background:**

Paternal mental health in pregnancy and postpartum has been increasingly highlighted as important both in its own right, but also as crucial for the development of children. Rates of help‐seeking among fathers is low, possibly due to conceptualising their own difficulties as stress rather than problems with mood. The relationship between paternal stress and child outcomes has not been investigated.

**Methods:**

This study used data from the Finnish CHILD‐SLEEP birth cohort. Data were available for 901 fathers and 939 mothers who completed questionnaires on demographics, stress, anxiety and depression at 32 weeks gestation, 3 months, 8 months and 24 months postpartum. Parental report of child emotional and behavioural problems was collected at 24 months.

**Results:**

Around 7% of fathers experienced high stress (over 90% percentile) at each timepoint measured in the perinatal period, rising to 10% at 2 years postpartum. Paternal stress measured antenatally, at 3 and 24 months was associated with child total problems at 24 months, while paternal depression and anxiety were not related to child outcomes when in the same model. After adjusting for concurrent maternal depression, anxiety and stress, an association remained between paternal stress at each timepoint and child total problem scores at 24 months. The strongest association was with paternal stress at 3 months (OR 3.17; 95% CI 1.63–6.16). There were stronger relationships between paternal stress and boys' rather than girls' total problem scores, although the interactions were not statistically significant.

**Conclusions:**

Paternal stress is an important manifestation of perinatal distress and is related to child mental health, particularly when present in the early postpartum months. Paternal stress should therefore be assessed in the perinatal period, which presents opportunities for early intervention and prevention of difficulties for both father and child.

## Introduction

The transition to parenthood can be difficult for parents and the exacerbation or onset of mental health problems are common in men and women. While the impact of maternal perinatal (during pregnancy and the postnatal year) mental health problems on children is well researched (Stein et al., 2014), paternal difficulties have received much less attention. Depression is thought to affect approximately 8% of fathers during the perinatal period (Cameron, Sedov, & Tomfohr‐Madsen, 2016), with anxiety symptoms also affecting 5%–10% of fathers (Philpott, Savage, Fitzgerald, & Leahy‐Warren, 2019; Simmonds et al., 2015).

There is increasing recognition that fathers' mental health is important not only in its own right, but in the distinct impact it may have on infant and child development (Cui et al., 2020; Sweeney & Macbeth, 2016). Fathers' antenatal anxiety and depression have been associated with postpartum parenting stress (Skjothaug, Smith, Wentzel‐Larsen, & Moe, 2018) and with negative impacts on emotional and behavioural outcomes in children at 2 months–7.5 years (Sweeney & Macbeth, 2016), but associations have not been found in all studies (Capron et al., 2015). Fathers' postnatal depressive symptoms have been associated with child emotional and behavioural problems at 3.5 years, and with a raised rate of conduct problems in boys (Ramchandani, Stein, Evans, & O'connor, 2005).

The impact of paternal depression on children may be mediated by a number of factors including couple relationship conflict (Gutierrez‐Galve, Stein, Hanington, Heron, & Ramchandani, 2015), negative parenting behaviours (Sweeney & Macbeth, 2016) or by a conflictual relationship with the child (Nath, Russell, Kuyken, Psychogiou, & Ford, 2016). The presence of paternal depression may compound the risks presented by maternal depression on children (Pietikainen et al., 2020). Early paternal parenting can be impacted by poor mental health; observational studies have found depressed fathers to be more withdrawn and less stimulating with their 3‐month infants, and this was associated with externalising behaviour problems at 1 year and cognitive development at 3 years (Ramchandani et al., 2013; Sethna et al., 2017). Fathers with anxiety symptoms encourage less exploration and challenge than non‐anxious controls in infants of 10–15 months, and this has been associated with increased infant anxiety (Moller, Majdandzic, & Bogels, 2015). Conversely, positive parenting and involvement by fathers have been shown to be associated with improved child outcomes (Shannon, Tamis‐Lemonda, London, & Cabrera, 2002).

Identification and help‐seeking in fathers with mental health problems is low (Call & Shafer, 2018). One reason is that fathers may experience and express psychological difficulties as stress rather than the emotional symptoms captured by most standardised measures (Darwin et al., 2017). Stress is the concept that environmental demands exceed an individual's adaptive capacity (Cohen, Janicki‐Deverts, & Miller, 2007), which may be triggered in parents by the combined demands of caregiving, financial provision and relationship changes. Measurement of perceived stress may therefore be important in understanding paternal difficulties and the impact on children.

Paternal mental health problems are often intricately bound to aspects of the family environment. Paternal and maternal depression are moderately correlated (Paulson & Bazemore, 2010) and may influence each other (Goodman, 2004; Paulson, Bazemore, Goodman, & Leiferman, 2016). Furthermore, marital distress has been associated with a twofold increase in paternal depression (Chhabra, Mcdermott, & Li, 2020). Parenting stress has also been associated with paternal depression and anxiety (Chhabra et al., 2020; Demontigny, Girard, Lacharité, Dubeau, & Devault, 2013). As in the case of maternal mental health difficulties (Glover, 2014), the nature and timing of paternal symptoms of anxiety, depression and stress may therefore have different effects on child outcomes, and this information would be important in determining the optimal time to screen and intervene.

### Aims of the study

The current analysis aimed to investigate the contributions of paternal stress, anxiety and depression from pregnancy through the postpartum, to child psychological outcomes at 2 years. To investigate the independent role of fathers, we controlled for measures of mother's emotional distress. We studied the impact of paternal factors at different timepoints during the perinatal period to assess their relative contribution. It was hypothesised that paternal emotional distress would have a unique impact on child outcomes over and above mother's stress, depression and anxiety. Finally, we examined whether the effects of paternal factors on child emotional problems are similar for boys and girls.

## Materials and methods

### Participants

This study uses data from the Finnish CHILD‐SLEEP birth cohort, a longitudinal cohort study; the study design is reported in detail elsewhere (Paavonen et al., 2017). The ethics committee board of Pirkanmaa Hospital District approved the CHILD‐SLEEP study protocol (R11032) and all participants gave their written informed consent. Parents were approached to participate during pregnancy during routine maternity appointments. Maternal and paternal questionnaires were completed at about gestational week 32 (1,667 women and 1,598 men), and postpartum at the 3‐month (1,432 women, 1,343 men), 8‐month (1,299 women and 1,211 men) and 2‐year (1,038 women, 776 men) time‐points. The participants were representative of the background population in terms of age and number of children (Paavonen et al., 2017).

At 2 years, data were available on 950 children, of whom we excluded five twins, five children with developmental disability or other severe syndromes and one child with too much missing information on the relevant measures leaving final sample of 939 children. The child questionnaire was completed by parents: approximately 69% of the child questionnaires by mothers, 30% by both parents together and 1% by fathers.

### Measures

#### Demographic information

Parental background information including age, education level (‘none or some vocational training’ vs. ‘vocational school or polytechnic’ vs. ‘university’), monthly income after taxes (‘<1,000€’ vs. ‘1,000–2,000€’ vs. ‘>2,000€’), number of other children in the family (‘0’ vs. ‘1’ vs. ‘two or more’) and smoking during pregnancy (‘at least once during last six months’ vs. ‘not at all’) were asked as part of the prenatal questionnaire. Birthweight, sex and birth date of the index child were collected from the hospital registers and information on daycare use was collected postnatally.

#### Standardised scales and missing items

For all standard scales up to 20% missing items were permitted, and missing items were replaced with the scale mean. The cut‐offs were defined based on prenatal timepoint and the same cut‐offs were used for each measurement point. The same cutoffs were used at all points for mothers and fathers.

#### Parental stress

Stress was measured by a 5‐item version of the Perceived Stress Scale (Cohen, Kamarck, & Mermelstein, 1983), each of the items scored on a 5‐point scale. The total score was calculated (range 0–20) after reverse coding two items. A cut‐off level of 10 or more (90th percentile) was used to indicate a ‘high’ level of stress. The included items were:

1. In the last month, how often have you felt that you were unable to control the important things in your life?
2. In the last month, how often have you felt confident about your ability to handle your personal problems? (reverse coded)
3. In the last month, how often have you felt that things were going your way? (reverse coded)
4. In the last month, how often have you felt difficulties were piling up so high that you could not overcome them?
5. In the last month, how often have you felt ‘stressed’?

#### Parental depression

Parental depressive symptoms were assessed by the 10‐item version of the Center for Epidemiological Studies Depression Scale, CES‐D (Irwin, Artin, & Oxman, 1999; Radloff, 1977). Example items were ‘I felt sad’, ‘I felt lonely’. Items are rated on a 4‐point scale and summed after reverse scoring two of the items (scale range 0–30, higher scores indicating more severe depressive symptoms). The cut‐off level of 10 or more points was used to indicate increased depressive symptoms (Grzywacz, Hovey, Seligman, Arcury, & Quandt, 2006).

#### Parental anxiety

Anxiety was assessed by using six items from the STAI anxiety scale (Bieling, Antony, & Swinson, 1998), with each item rated on a 4‐point scale. Example items were ‘I feel nervous and restless’, ‘Some important thought runs through my mind and bothers me’. A total score consisted of summed items (scale range 6–24). The sum score was dichotomised at prenatal 90^th^ percentile (12 points or more) to indicate increased anxiousness (Kiviruusu et al., 2020; Paavonen et al., 2017). Because there was an overlapping item in the stress and anxiety scales, we conducted sensitivity analyses, where the STAI score was calculated without this item and then replicated all the models with this variable. We found that the results did not change (data not presented).

#### Children's emotional problems at the age of 2

The Brief Infant‐Toddler Social and Emotional Assessment (BITSEA) was used to assess children's social‐ and emotional problems as well as competencies (Briggs‐Gowan, Carter, Irwin, Wachtel, & Cicchetti, 2004). Each item was rated on a 3‐point scale: ‘Not true/rarely (=0)’ versus ‘Somewhat true/sometimes (=1)’ versus ‘Very true/often (=2)’.

The problems scale consists of 31 items (scale range 0–62) and comprises subscales of externalising (e.g. overactivity, aggression and defiance, 7 items, scale range 0–14) and internalising problems (e.g. symptoms of depression and anxiety, 14 items, scale range 0–28) as well as items concerning dysregulation, maladaptive and atypical behaviours. Sum scores were calculated by adding the items together; higher problem scores indicate higher risk for social–emotional problems. The cut‐off ≥14 for the total problem scale was selected based on Finnish and international studies (Alakortes, Fyrstén, Carter, Moilanen, & Ebeling, 2015; Briggs‐Gowan, Carter, Irwin, Wachtel, & Cicchetti, 2004). For the externalising and internalising problem subscales, the highest 25th percentiles, ≥5 and ≥5.5 points, respectively, were selected as cut‐offs (Briggs‐Gowan & Carter, 2008). The use of dichotomised variables was to indicate a clinically meaningful group of children with elevated symptoms.

### Drop‐out

When comparing parental prenatal measures between participants and non‐participants at 24 months follow‐up, it was found that among both fathers and mothers younger age, less education, lower income and more previous children, and among fathers also smoking during pregnancy, were associated with non‐participation (*p* < .05). Mothers that were more stressed, depressed or anxious antenatally were more likely to drop‐out at 24 months (*p* < .01), while among fathers none of these measures were associated with non‐participation at 24 months (*p* > .10). For the 939 children included in the present study, there were 901 fathers and 939 mothers with at least some relevant data available for the present analyses; using listwise deletion valid *N* varied in the analyses according to the measure and timepoint (see Table 1).

**Table 1:** Characteristics of the fathers and mothers

Measure		N	Fathers (*N* = 901)	N	Mothers (*N* = 939)
			Mean (*SD*)/% (*N*)		Mean (*SD*)/% (*N*)
Age	Prenatally	826	32.9 (4.85)	930	31.1 (4.36)
Previous children	0	793	48.9% (388)	877	49.9% (438)
	1		33.4% (265)		33.8% (296)
	≥2		17.7% (140)		16.3% (143)
Education (vocational)	None/Some vocational training	872	9.5% (83)	918	4.4% (40)
	Vocational school/Polytechnic		56.5% (493)		56.6% (520)
	University		33.9% (296)		39.0% (358)
Monthly income during pregnancy	<1,000€	882	6.1% (54)	923	20.9% (193)
	1,000–2,000€		31.7% (280)		50.9% (470)
	>2,000€		62.1% (548)		28.2% (260)
Smoking during pregnancy (any)		889	28.7% (255)	936	4.8% (45)
Stress, Cohen ≥10	Prenatally	892	7.8% (70)	937	7.8% (73)
	3 months	841	7.4% (62)	900	11.0% (99)
	8 months	826	7.4% (61)	896	12.9% (116)
	24 months	695	10.1% (70)	904	15.3% (138)
Depression, CES‐D ≥ 10	Prenatally	890	4.3% (38)	935	9.7% (91)
	3 months	844	4.7% (40)	900	9.7% (87)
	8 months	839	7.3% (61)	893	14.2% (127)
	24 months	696	10.5% (73)	904	15.2% (137)
Anxiety, STAI ≥12	Prenatally	892	10.8% (96)	936	11.6% (109)
	3 months	841	9.3% (78)	900	14.2% (128)
	8 months	838	7.3% (61)	892	15.1% (135)
	24 months	694	11.8% (82)	904	16.6% (150)

### Statistical analyses

Descriptive statistics for parental stress, depression and anxiety at each timepoint were calculated first.

Logistic regression analyses were conducted using dichotomised BITSEA total problem, externalising and internalising problems scores as dependent variables and dichotomised paternal stress, depression and anxiety sum scores at each timepoint as independent variables. This approach, i.e., using dichotomised variables, was chosen to (i) focus on clinically meaningful scores according to the study question and (ii) to avoid violations in normality assumptions related to linear models. Separate analyses were conducted for the four timepoints. The analyses were adjusted first for (a) child's age, sex, birth weight, paternal age, education, number of other children in the family, and then in addition also for (b) concurrent paternal stress, depression and anxiety (i.e., all three measures from the given timepoint simultaneously in the models).

In order to investigate whether paternal stress had an independent association with children's emotional problems over and above maternal contributions, we adjusted the logistic regression analyses of paternal stress for (a) concurrent maternal stress (i.e., from the same timepoint as the father's stress) and then also for (b) concurrent maternal depression and anxiety at each time‐point. These analyses were conducted for the total sample and for boys and girls separately. Interactions with paternal stress and child sex were investigated in terms of BITSEA total, externalising and internalising problems in logistic regression. These analyses were adjusted for child's age, sex, birth weight, paternal age, education and number of other children in the family.

In a logistic regression model, we analysed the relative importance of father's stress at the four timepoints when predicting child's elevated BITSEA total score and calculated the correlations between the four stress measures. We constructed classes of comorbidities from father's distress measures and cross‐tabulated these against child's elevated BITSEA total score. Finally, we ran a stepwise logistic regression model (forward; conditional on model improvement) with all father's and mother's distress variables from all four timepoints and all background variables as candidate predictors of child's elevated BITSEA total score at 24 months.

## Results

### Characteristics of participants

Table 1 presents data on parents' socio‐demographic characteristics and the prevalence rates of stress, depression and anxiety at each timepoint. Generally, more mothers than fathers scored above the threshold on the three distress measures during the study period, while the prevalence rate among both mothers and fathers on each measure was highest at 24 months. The proportion of high‐stress fathers remained relatively consistent between 7%–8% until an increase at 2 years postpartum when the rate was 10.1%. Stress, depression and anxiety were inter‐correlated in both fathers (0.35–0.57) and mothers (0.41–0.64) at all time points. 28.6% of fathers with high stress (prenatally) did not have any comorbidity, with 2.9% experiencing concurrent depression and 37.1% concurrent anxiety and 31.4% both depression and anxiety.

The sample of children were 52.9% male and 48.7% were not in daycare (Table 2). At 2 years, 18.3% of the children scored above the threshold for problems on the BITSEA total score.

**Table 2:** Characteristics of the children

Measure		All (*N* = 939)	Boys (*N* = 497)	Girls (*N* = 442)
		Mean (*SD*)/% (*N*)	Mean (*SD*)/% (*N*)	Mean (*SD*)/% (*N*)
Age (months)		24.2 (1.43)	24.3 (1.39)	24.2 (1.47)
Gestational weight (g)		3,575 (451.5)	3,635 (455.8)	3,508 (437.4)
Daycare at 24 months	No	48.7% (455)	52.7% (261)	44.1% (194)
	Part‐time	17.0% (159)	16.0% (79)	18.2% (80)
	Full‐time	34.3% (321)	31.3% (155)	37.7% (166)
Total problems (BITSEA)	Mean (*SD*)	9.19 (5.27)	9.35 (5.23)	9.02 (5.32)
	≥14 p	18.3% (169)	18.9% (92)	17.7% (77)
Externalising problems	Mean (*SD*)	3.09 (2.17)	3.35 (2.18)	2.80 (2.12)
	≥5 p	23.9% (221)	28.0% (137)	19.3% (84)
Internalising problems	Mean (*SD*)	3.73 (2.87)	3.59 (2.87)	3.88 (2.87)
	≥5.5 p	24.9% (229)	22.0% (107)	28.2% (122)

### Influence of paternal distress on child's emotional problems

In the first set of analyses, adjusted for father's age, education and previous children and child's sex, age and birthweight, only paternal antenatal depression was not related to child problem scores at 2 years, with paternal stress and anxiety at each timepoint strongly related to child problems at 2 years.

Paternal stress at each timepoint was the most strongly associated paternal distress variable, with ORs ranging from 2.88 at 8 months to 4.25 at 3 months (Table 3). Also, paternal depression (OR 4.14) and anxiety (OR 2.65) measured at 3 months postpartum had their strongest associations with child's total problems at 24 months. Moreover, when the stress variables from four timepoints were considered simultaneously, the 3‐month stress variable remained as the only significant predictor of elevated child total BITSEA score (Table S1). This indicates that, while the inter‐correlations of the stress variables ranged between 0.35 and 0.48 (Table S2), the effect of father's stress at 3 months is not completely mediated by stress reported at later timepoints.

**Table 3:** Paternal stress, depression and anxiety predicting child's total, externalising and internalising problems at age two (adjusted logistic regression analyses)

	Total problems				Externalising problems				Internalising problems			
Paternal symptoms	AOR^a^ (95% CI)	*p*	AOR^b^ (95% CI)	*p*	AOR^a^ (95% CI)	*p*	AOR^b^ (95% CI)	*p*	AOR^a^ (95% CI)	*p*	AOR^b^ (95% CI)	*p*
Gw 32												
Stress	**3.06 (1.72–5.45)**	**<.001**	**3.19 (1.52–6.68)**	**.002**	1.27 (0.69–2.35)	.439	1.58 (0.74–3.35)	.238	**1.80 (1.01–3.23)**	**.048**	1.71 (0.83–3.51)	.143
Depression	1.59 (0.65–3.85)	.307	0.65 (0.23–1.87)	.424	1.15 (0.49–2.67)	.751	1.19 (0.45–3.15)	.733	1.44 (0.61–3.38)	.405	0.95 (0.36–2.54)	.918
Anxiety	**1.87 (1.09–3.20)**	**.023**	1.17 (0.58–2.35)	.660	0.83 (0.47–1.45)	.506	0.64 (0.32–1.28)	.203	1.43 (0.85–2.40)	.180	1.14 (0.60–2.16)	.685
3 months												
Stress	**4.25 (2.28–7.90)**	**<.001**	**2.47 (1.10–5.56)**	**.029**	**2.14 (1.15–3.99)**	**.017**	2.13 (0.96–4.75)	.065	**3.41 (1.85–6.28)**	**<.001**	**2.90 (1.34–6.27)**	**.007**
Depression	**4.14 (2.01–8.51)**	**<.001**	2.08 (0.86–5.03)	.105	1.97 (0.94–4.14)	.072	1.60 (0.66–3.88)	.301	1.98 (0.94–4.18)	.074	0.86 (0.34–2.17)	.742
Anxiety	**2.65 (1.49–4.71)**	**.001**	1.44 (0.70–2.93)	.319	1.25 (0.69–2.28)	.456	0.78 (0.38–1.61)	.509	**2.11 (1.21–3.69)**	**.009**	1.38 (0.71–2.68)	.348
8 months												
Stress	**2.88 (1.55–5.37)**	**.001**	2.09 (0.95–4.63)	.068	1.07 (0.55–2.09)	.836	1.44 (0.63–3.32)	.387	1.63 (0.87–3.06)	.130	0.98 (0.44–2.17)	.951
Depression	**2.72 (1.44–5.16)**	**.002**	2.02 (0.91–4.51)	.086	1.07 (0.55–2.09)	.843	1.59 (0.69–3.65)	.275	**2.53 (1.37–4.68)**	**.003**	**2.41 (1.13–5.12)**	**.022**
Anxiety	**2.20 (1.16–4.16)**	**.015**	1.03 (0.42–2.52)	.947	0.69 (0.34–1.42)	.313	0.39 (0.14–1.06)	.064	1.83 (0.99–3.38)	.053	1.26 (0.56–2.85)	.583
24 months												
Stress	**3.04 (1.65–5.60)**	**<.001**	**2.41 (1.11–5.22)**	**.027**	1.06 (0.55–2.03)	.866	1.04 (0.46–2.32)	.929	**2.29 (1.27–4.16)**	**.006**	1.87 (0.90–3.89)	.096
Depression	**1.97 (1.07–3.65)**	**.031**	0.97 (0.43–2.16)	.932	1.16 (0.63–2.16)	.628	1.26 (0.59–2.67)	.547	1.72 (0.96–3.08)	.067	1.07 (0.52–2.22)	.856
Anxiety	**2.41 (1.35–4.30)**	**.003**	1.61 (0.78–3.35)	.201	0.94 (0.51–1.76)	.849	0.83 (0.39–1.77)	.624	**1.91 (1.09–3.33)**	**.024**	1.40 (0.71–2.75)	.332

^a^ Adjusted for child's age at 24 months, sex, birth weight, paternal age, paternal education and number of previous children.

^b^ Adjusted for child's age at 24 months, sex, birth weight, paternal age, paternal education, number of previous children and concurrent paternal stress, depression and anxiety (all simultaneously in the model).

Bold values indicates p < 0.05.

After adjusting the models further for concurrent paternal stress, depression and anxiety, only paternal stress measured antenatally (OR 3.19), at 3 months (OR 2.47) and 24 months (OR 2.41) was significantly associated with child problems at 24 months (Table 3). When analysing the relative contributions of father's stress, depression and anxiety as well as their comorbidities, elevated child symptom scores at 24 months were found in 45% of children of men with high stress only (prenatally), compared with 31.3% of those having stress with comorbid depression and/or anxiety, 19.1% of those with depression and/or anxiety without stress and 17.1% of those with no high symptoms (Table S3).

**Figure 1:**
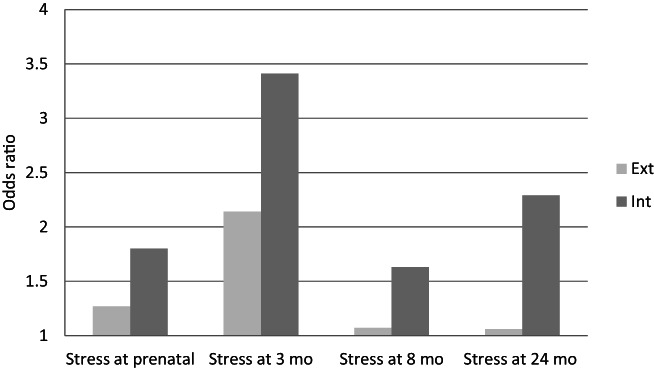
Odds ratios for paternal stress from late pregnancy until 24 months after delivery predicting children's externalising and internalising problems at 24 months

Examining the BITSEA subscales, there was a more consistent pattern of associations with child internalising than externalising problems, with paternal stress variable most often associated with internalising problems in the first set of adjusted models, again strongest at 3 months (Table 3; Figure 1).

### Influence of paternal stress taking maternal distress into account

Given the prominence of paternal stress, we conducted a further logistic regression to examine its unique contribution, adjusting (in addition to characteristics of the child and father) first for maternal stress and then also with mother's anxiety and depression (Table 4). Paternal stress at each of the measurement points from pregnancy remained strongly associated with child total problems at 2 years, after adjusting for all the mother's distress factors (ORs 2.34–3.17). In a supplementary analysis including all father's and mother's distress variables from all timepoints, again the role of father's stress and the 3‐month timepoint was emphasised (Table S4a,b).

**Table 4:** Paternal stress predicting child's total, externalising and internalising problems at age two adjusting for maternal stress, depression and anxiety

	Total problems				Externalising problems				Internalising problems			
	AOR^a^ (95% CI)	*p*	AOR^b^ (95% CI)	*p*	AOR^a^ (95% CI)	*p*	AOR^b^ (95% CI)	*p*	AOR^a^ (95% CI)	*p*	AOR^b^ (95% CI)	*p*
Total												
Paternal stress												
32 gw	**2.76 (1.53–4.98)**	**.001**	**2.66 (1.47–4.83)**	**.001**	1.14 (0.61–2.15)	.677	1.16 (0.61–2.18)	.655	1.63 (0.90–2.97)	.108	1.52 (0.83–2.80)	.178
3 months	**3.52 (1.85–6.69)**	**<.001**	**3.17 (1.63–6.16)**	**.001**	**1.97 (1.04–3.74)**	**.037**	1.83 (0.95–3.51)	.069	**2.92 (1.56–5.49)**	**.001**	**2.65 (1.39–5.05)**	**.003**
8 months	**2.43 (1.28–4.65)**	**.007**	**2.34 (1.21–4.51)**	**.011**	0.97 (0.50–1.91)	.936	0.93 (0.47–1.85)	.841	1.43 (0.75–2.73)	.279	1.36 (0.70–2.63)	.362
24 months	**2.88 (1.55–5.36)**	**.001**	**2.52 (1.33–4.80)**	**.005**	0.97 (0.50–1.89)	.930	0.85 (0.42–1.69)	.635	**2.10 (1.14–3.85)**	**.017**	1.79 (0.95–3.38)	.071
Boys												
Paternal stress												
32 gw	**2.96 (1.24–7.05)**	**.014**	**3.14 (1.29–7.63)**	**.011**	1.30 (0.55–3.04)	.552	1.37 (0.58–3.25)	.469	2.29 (0.95–5.47)	.064	2.27 (0.93–5.54)	.071
3 months	**4.59 (1.81–11.63)**	**.001**	**4.77 (1.83–12.45)**	**.001**	2.20 (0.92–5.28)	.078	2.17 (0.87–5.14)	.098	**2.62 (1.04–6.59)**	**.041**	**2.65 (1.05–6.74)**	**.040**
8 months	1.85 (0.71–4.82)	.206	1.85 (0.70–4.94)	.218	0.83 (0.34–2.03)	.685	0.83 (0.38–2.02)	.675	0.69 (0.24–2.02)	.498	0.67 (0.23–1.99)	.474
24 months	**3.41 (1.46–7.99)**	**.005**	**2.96 (1.24–7.08)**	**.015**	0.83 (0.35–1.96)	.663	0.74 (0.30–1.81)	.510	2.00 (0.83–4.83)	.124	1.70 (0.68–4.23)	.257
Girls												
Paternal stress												
32 gw	**2.46 (1.06–5.72)**	**.037**	2.19 (0.93–5.18)	.074	0.86 (0.32–2.26)	.754	0.76 (0.28–2.04)	.580	1.30 (0.56–3.01)	.538	1.18 (0.47–2.67)	.804
3 months	2.37 (0.91–6.16)	.077	1.76 (0.62–4.98)	.285	1.76 (0.65–4.76)	.265	1.69 (0.59–4.79)	.327	**3.12 (1.26–7.71)**	**.014**	2.34 (0.89–6.10)	.084
8 months	**3.25 (1.30–8.17)**	**.012**	**3.42 (1.31–8.97)**	**.012**	1.05 (0.36–3.04)	.930	1.08 (0.36–3.26)	.892	**2.59 (1.06–6.35)**	**.037**	**2.71 (1.06–6.94)**	**.037**
24 months	**2.88 (1.11–7.52)**	**.031**	2.46 (0.89–6.79)	.082	1.17 (0.39–3.45)	.780	0.95 (0.31–2.95)	.635	**2.79 (1.14–6.85)**	**.025**	2.49 (0.97–6.39)	.057

^a^ Adjusted for child's age at 24 months, sex, birth weight, paternal age, paternal education, number of previous children and mother's concurrent stress.

^b^ Adjusted for child's age at 24 months, sex, birth weight, paternal age, paternal education, number of previous children and mother's concurrent stress, depression and anxiety.

In terms of subscales with all factors adjusted for, there were no significant associations between paternal stress and externalising problems, but paternal stress scores at 3 months (OR 2.65) were independently associated with child internalising problems. In the fully adjusted models, paternal stress measured in pregnancy, at 3 and 24 months was associated with total problem scores in boys, but only at 8 months in girls (Table 4). After all adjustments only stress measured at 3 months in boys and at 8 months in girls was associated with child's internalising problems at 24 months. There was no interaction between child's sex and paternal stress as all interaction terms child sex × paternal stress on BITSEA scores were non‐significant (*p* > .05) at all timepoints.

## Discussion

This study aimed to investigate the potential role of perinatal paternal distress including stress in child outcomes at 2 years. Our results showed that stress, measured at various timepoints from pregnancy onwards, made a unique contribution over and above other measurements of paternal mental health despite statistically taking into account maternal depression, anxiety and stress. Paternal stress measured at 3 months had the most consistent associations with child problems at 2 years.

The mental health of fathers has received increased attention in recent years, and this study adds to a body of research highlighting the unique contribution of paternal wellbeing to child outcomes (Sweeney & Macbeth, 2016). Our study highlighted associations of paternal stress from pregnancy onwards on child problems, and with greater influence on child internalising than externalising symptoms. Previous research has also found associations between prenatal paternal distress (including anxiety and depression symptoms) and child outcomes (Kvalevaag et al., 2013). The mechanisms of influence of paternal stress on child outcomes are not yet clear and may include emotional or physical unavailability with the child, negative parenting style, relationship conflict, impact on maternal mental health or a combination of these. In one large study, the impact of paternal depression on maternal mental health and marital conflict played a key role in child outcomes (Gutierrez‐Galve et al., 2015). There may also be a number of wider mechanisms. Paternal distress may impact the child's environment and therefore development in several ways – engagement with protective factors such as supportive family or community, adverse impacts on occupational functioning, status and income. Genetic effects and gene–environment interactions may also play a role with parents with mental disorder more likely to experience social adversity in addition to genetic associations (Ramchandani & Psychogiou, 2009).

Although the early months of caregiving are known to be challenging, reflected in relatively high paternal distress of all types at 3 months, our results also highlight that maternal and paternal stress does not diminish over time but seems to increase after the end of the traditional ‘perinatal period’ and were at their highest at 2 years postpartum. Even so, stress at 3 months appears to have the most impact on child outcomes at 2 years. Previous research has found that poor job quality, poor relationship quality, maternal distress and low parental self‐efficacy contribute to father's perinatal mental distress (Giallo et al., 2013). These factors may be particularly concentrated at 3 months, with paternal negative birth experiences also playing a role (Gawlik et al., 2014; Schobinger, Stuijfzand, & Horsch, 2020). At this stage infant sleep difficulties and associated parental sleep debt may be particularly difficult for fathers returning to work. These converging factors may also contribute to stress at 2 years postpartum, potentially combined with the context of trying for further pregnancies. The timing of shared parental leave may explain the lower rates of psychopathology in the early perinatal period and the increased stress at 2 years as it ends. For some, the parenting of increasingly autonomous and mobile 2‐year olds may be more challenging than the early stages and so the influence on parental mental health may be bi‐directional. Where children are experiencing difficulties, they may be becoming apparent around this stage as milestones are not met (Barroso, Mendez, Graziano, & Bagner, 2018). For parents already experiencing stress these emergent difficulties may be particularly difficult to manage, or potentially such parents may perceive child behaviour as more problematic.

The concept of parenting stress, i.e., stress specifically related to parenting, can interact with the emergence and maintenance of child behavioural problems (Neece, Green, & Baker, 2012). Our data indicate that parental stress measured in pregnancy and especially the early postpartum may play a role. Maternal antenatal distress has been found to exert a long‐lasting effect on child emotional outcomes, possibly due to the impact on stress responsivity and the HPA axis (Braithwaite et al., 2017; Capron et al., 2015; Werner et al., 2013). During pregnancy, cortisol responses can be elevated in fathers where there is high maternal distress (Braren, Brandes‐Aitken, Ribner, Perry, & Blair, 2020), again highlighting the interplay between the mental health of parents. Pregnancy and early postpartum may be the most demanding stage of adaptation to parenting an infant, even for experienced parents who may be more used to the role but are managing the care of multiple children. Previous studies have not found differences in depressive symptoms between first‐time fathers and others (Wells & Aronson, 2021). Approximately half of the sample were first‐time parents, and family size was adjusted for in the analysis. The relationship with paternal stress remained after adjusting for number of other children.

The unique contribution of perinatal paternal stress has implications for approaching and intervening with fathers. Men may be reluctant to seek help or express their needs during this time and may feel excluded from the maternal focus of general obstetric and specialist perinatal mental health services (Darwin et al., 2017; Seidler, Dawes, Rice, Oliffe, & Dhillon, 2016). Explicit effort may be required to engage fathers in discussions about the support they may need. Contact initiated in pregnancy by healthcare providers could be helpful in preventing or ameliorating parental and child symptoms (Wells & Aronson, 2021) and mothers might be able to provide information on possible difficulties in partners during these contacts, for example using the EPDS‐partner version in order to direct fathers to counselling or other help (Fisher et al., 2021). In the UK, it is recommended that partners of mothers in contact with perinatal mental health services and maternity outreach clinics are invited for screening (NHS, 2019) and various organisations including the US Preventive Services Task Force have provided guidelines for paternal depression identification and early intervention. Recent professional consensus indicates that interventions for depression should be tailored to men and framed around fatherhood rather than mental health (Domoney, Trevillion, & Challacombe, 2020). While mood symptoms are important, our study highlights the need to frame paternal mental wellbeing more broadly than symptoms of anxiety and depression. Fathers may under report symptoms of depression such as sadness due to cultural conceptions of masculinity and may be more likely to use avoidance, escape or numbing from emotional distress (Fisher et al., 2021). Stress may signify risk for future mood disorders, for example via the impact on sleep (González‐Mesa et al., 2019) and is therefore a useful target for screening and intervention.

Orienting intervention around stress/confidence may increase paternal engagement (Primack, Addis, Syzdek, & Miller, 2010). A better understanding of the causes of paternal stress may assist the development of targeted intervention and prevention strategies that could benefit fathers and their children. Interventions around workplace flexibility, peer support from other fathers, family therapy and innovative approaches such as tailored mobile phone apps and football teams (recently launched for bereaved fathers) to connect may be helpful to navigate fathers' reticence to engage with mental health services (Domoney, Iles, & Ramchandani, 2018). Given the strong relationships between maternal and paternal mental health, the whole family needs to be considered. Given our results strongly emphasise the 3‐month timepoint, the early postpartum months might be a good point to intervene.

Existing research on differential gender effects has tended to focus on maternal mental health. For example, De Bruijn, Van Bakel, and Van Baar (2009) found maternal emotional disturbances in early pregnancy to more strongly affect boys, whereas girls were affected later during pregnancy (de Bruijn et al., 2009; Sandman, Glynn, & Davis, 2013). We did not find an interaction between child sex and paternal stress, with impacts on both girls and boys but at different timepoints. Future studies could consider the inter‐relationships between maternal and paternal health, their impact on each other and the potential effects on children of these inter‐relationships. Alternative statistical models could assess the relative impact of maternal and paternal trajectories of distress on child outcomes and ideally should also take into account important factors related to child–parent interaction, marital conflict and child health. Furthermore, factors beyond paternal stress related to family and paternal wellbeing and functioning should be incorporated.

### Strengths and limitations

A strength of the study was that it was able to recruit and retain a large sample of mothers and fathers over a 2‐year period. There was some attrition over time but this did not appear to be related to paternal mental health. However, younger parents with more children and mothers with higher stress were more likely to drop out, possibly impacting on the contribution of maternal stress to outcomes in this analysis. The questionnaire battery utilised shorter versions of some measures, and within‐study cutoffs were used to establish elevated levels of anxiety, depression and stress which may limit the generalisability of findings. As the same cutoffs were used for men and women, it is possible that some depressed fathers would only have endorsed some of the items and therefore did not reach ‘caseness’ despite being true cases. It has been indicated that fathers may have a lower EPDS score than mothers to signify ‘caseness’ (Edmondson, Psychogiou, Vlachos, Netsi, & Ramchandani, 2010). If fathers underreport depressive symptoms, the use of dichotomised scores could therefore have possibly underestimated the effect of paternal depression in the analysis. Indeed, this might suggest that in addition to the more traditional measures of depression and anxiety, a screener for stress might have clinical utility, and in our analyses it seems to have predictive value for child psychiatric outcomes.

All measures of parental distress made contributions to child outcomes suggesting they all are relevant in terms of child development. The issue of possible overlap between constructs needs to be considered; the correlations were generally moderate, with one strong. The wording of one item in the stress scale overlapped to a considerable extent with the anxiety scale (use of the phrase ‘things piling up high’ in both scales), but sensitivity analyses showed that this did not affect the results. However, while there was comorbidity between stress, depression and anxiety, our data indicated that father's stress has an independent role (i.e., whether or not presenting with comorbid depression and/or anxiety) relative to child emotional symptoms at 24 months.

Child data were from parent report which may have introduced bias, although regarding the father, the risk of bias was reduced as parental report on the child was by the mother alone (in 69% of the cases) or mother and father together (30%). The associations between child problems and parental distress may also be reciprocal, particularly at later timepoints, which should be kept in mind when interpreting the results. However, prenatally reported stress scores are independent of the child's behaviour. Moreover, some shared variance is related to persistent stress (i.e. the correlations across the timepoints). It is possible that there are genetic factors influencing the associations, and the expression of distress in both child and parent, which this study did not measure. It was not possible to conduct clinical interviews to establish levels of distress or child symptoms.

## Conclusions

One of the most compelling of our results was the robust effect of paternal stress over and above paternal depression and anxiety. This emphasises and further elaborates the idea that for a father it is not necessarily mood symptoms that should be targeted for screening in the perinatal period. Furthermore, we also found that paternal stress had an independent effect on child's emotional problems after accounting for maternal stress, depression and anxiety. This result is especially interesting given our earlier findings suggesting that maternal, not paternal depression is of importance in relation to child's emotional problems (Pietikainen et al., 2020). Our present results now complement this picture, suggesting that paternal stress, with its independent effect on child outcomes, is a good candidate for more relevant measures of screening during pregnancy and postpartum. Support offered in the early postpartum may be most impactful in ameliorating the impact on fathers and children, although fathers should be screened at other timepoints for their own wellbeing, given our finding that greater numbers of fathers may be experiencing stress 2 years after birth than in the early stages.

**Key points**

- Paternal mental health has an impact on children.
- Fathers do not readily seek help in the perinatal period and may find the focus on depression and anxiety less relevant to them.
- Paternal stress, particularly measured at 3m postpartum, is more strongly related to child outcomes than depression or anxiety.
- Paternal stress makes a unique contribution to child outcomes after taking maternal mental health into account.
- Fathers should be screened for perinatal stress using tailored measures.

## Supporting information


**Table S1.** Father's stress at the four timepoint predicting child's elevated BITSEA total score at 24 months.
**Table S2.** Correlations (Spearman's rho) between father's stress measures from the four timepoints.
**Table S3.** Percentages of children with elevated BITSEA total score at 24 months by comorbidities of father's stress, depression and anxiety at different timepoints.
**Table S4.** (a) Results from a stepwise (forward conditional) logistic regression model with all father’s and mother’s distress variables from all four timepoints and all background variables as candidate predictors to be selected to the model predicting child’s elevated BITSEA total score at 24 months. Selected variables to the model in each step. (b) Results from a stepwise (forward conditional) logistic regression model (see Table S4a). Variables not entered to the model after final step (step 2).
