# Supplementary material for: Paternal perinatal stress is associated with children's emotional problems at 2 years
Source: J Child Psychol Psychiatry. 2022 Oct 10;64(2):277–88. doi: 10.1111/jcpp.13695 (PMC10092317; doi:10.1111/jcpp.13695)
Supplement: Supplementary file 1 — Table S1. Father's stress at the four timepoint predicting child's elevated BITSEA total score at 24 months. Table S2. Correlations (Spearman's rho) between father's stress measures from the four timepoints. Table S3. Percentages of children with elevated BITSEA total score at 24 months by comorbidities of father's stress, depression and anxiety at different timepoints. Table S4. (a) Results from a stepwise (forward conditional) logistic regression model with all father’s and mother’s distress variables from all four timepoints and all background variables as candidate predictors to be selected to the model predicting child’s elevated BITSEA total score at 24 months. Selected variables to the model in each step. (b) Results from a stepwise (forward conditional) logistic regression model (see Table S4a). Variables not entered to the model after final step (step 2). [file JCPP-64-277-s001.docx]

**Supporting Information**

**Table S1.** Father’s stress at the four timepoint predicting child’s elevated BITSEA total score at 24 months.

|  | Unadjusted models^a^ | | Adjusted model 1^b^ | | Adjusted model 2^c^ | |
| --- | --- | --- | --- | --- | --- | --- |
| Father’s stress (Cohen) | OR (95% CI) | p | OR (95% CI) | p | OR (95% CI) | p |
| prenatally | 2.71 (1.59-4.61) | <0.001 | 1.09 (0.46-2.59) | 0.851 | 1.31 (0.51-3.42) | 0.576 |
| 3 months | 3.59 (2.06-6.25) | <0.001 | 2.90 (1.28-6.55) | 0.011 | 2.98 (1.15-7.74) | 0.025 |
| 8 months | 2.39 (1.34-4.26) | 0.003 | 1.36 (0.57-3.22) | 0.490 | 1.38 (0.54-3.51) | 0.501 |
| 24 months | 2.76 (1.59-4.78) | <0.001 | 1.56 (0.79-3.10) | 0.201 | 1.57 (0.71-3.44) | 0.263 |
| Nagelkerke R Square |  |  | 0.053 | | 0.102 | |

^a^ Separate model for each timepoint; father’s stress as the only independent variable in the model

^b^ All stress variables from the four timepoints simultaneously in the model

^c^ Model 1 + child’s age at 24 months, sex, birth weight, paternal age, paternal education, number of previous children.

**Table S2.** Correlations (Spearman’s rho) between father’s stress measures from the four timepoints.

| Father’s stress (Cohen) | prenatally | | 3 months | | 8 months | |
| --- | --- | --- | --- | --- | --- | --- |
|  | rho | p | rho | p | rho | p |
| prenatally | - |  |  |  |  |  |
| 3 months | 0.480 | <0.001 | - |  |  |  |
| 8 months | 0.429 | <0.001 | 0.428 | <0.001 | - |  |
| 24 months | 0.364 | <0.001 | 0.372 | <0.001 | 0.347 | <0.001 |

**Table S3.** Percentages of children with elevated BITSEA total score at 24 months by comorbidities

of father’s stress, depression and anxiety at different timepoints.

|  | No stress/  No Depression/  No anxiety- | No, stress-  depression+ and/or  anxiety+^a^ | Stress only (no+  depression- or  anxiety- | stress+ with  depression+ and/or  anxiety+^b^ | Significance of Chi-square test (df=3) |
| --- | --- | --- | --- | --- | --- |
|  | % (N) | % (N) | % (N) | % (N) | p |
| prenatally | 17.1% (129) | 19.3% (11) | 45.0% (9) | 31.3% (15) | 0.002 |
| 3 months | 15.9% (114) | 22.6% (12) | 43.8% (7) | 38.1% (16) | <0.001 |
| 8 months | 16.9% (121) | 24.4% (11) | 27.8% (5) | 35.0% (14) | 0.014 |
| 24 months | 16.2% (90) | 20.6% (14) | 36.8% (7) | 34.0% (16) | 0.003 |

^a^ Depression and/or anxiety without stress

^b^ Depression and/or anxiety with stress

**Table S4a.** Results from a stepwise (forward conditional^a^) logistic regression model with all father’s and mother’s distress variables from all four timepoints and all background variables as candidate predictors to be selected to the model predicting child’s elevated BITSEA total score at 24 months. Selected variables to the model in each step.

| Model / Variables | OR (95% CI) | p | Nagelkerke  R Square |
| --- | --- | --- | --- |
| Step 1 |  |  |  |
| Father’s stress at 3 months | 4.68 (2.27-9.65) | <0.001 | 0.052 |
| Step 2 |  |  |  |
| Father’s stress at 3 months | 3.98 (1.89-8.38) | <0.001 | 0.074 |
| Mother’s anxiety at 3 months | 2.37 (1.28-4.39) | 0.006 |  |

^a^ Entry to the model conditional on significant (p < 0.05) change in model log-likelihood.

**Table S4b.** Results from a stepwise (forward conditional) logistic regression model (see Table S4a). Variables not entered to the model after final step (step 2).

| Variable | Score^a^ | df | p |
| --- | --- | --- | --- |
| Father’s stress, prenatal | 1.025 | 1 | 0.311 |
| Father’s stress, 8 months | 0.761 | 1 | 0.383 |
| Father’s stress, 24 months | 2.118 | 1 | 0.146 |
| Father’s depression, prenatal | 2.015 | 1 | 0.156 |
| Father’s depression, 3 months | 2.092 | 1 | 0.148 |
| Father’s depression, 8 months | 0.092 | 1 | 0.761 |
| Father’s depression, 24 months | 0.101 | 1 | 0.750 |
| Father’s anxiety, prenatal | 0.004 | 1 | 0.950 |
| Father’s anxiety, 3 months | 0.000 | 1 | 1.000 |
| Father’s anxiety, 8 months | 0.003 | 1 | 0.958 |
| Father’s anxiety, 24 months | 0.046 | 1 | 0.830 |
| Mother’s stress, prenatal | 0.627 | 1 | 0.429 |
| Mother’s stress, 3 months | 1.246 | 1 | 0.264 |
| Mother’s stress, 8 months | 3.369 | 1 | 0.066 |
| Mother’s stress, 24 months | 0.039 | 1 | 0.844 |
| Mother’s depression, prenatal | 0.467 | 1 | 0.494 |
| Mother’s depression, 3 months | 1.650 | 1 | 0.199 |
| Mother’s depression, 8 months | 0.003 | 1 | 0.959 |
| Mother’s depression, 24 months | 0.678 | 1 | 0.410 |
| Mother’s anxiety, 8 prenatal | 0.468 | 1 | 0.494 |
| Mother’s anxiety, 8 months | 0.085 | 1 | 0.770 |
| Mother’s anxiety, 24 months | 1.461 | 1 | 0.227 |
| Child’s age at 24 months | 1.391 | 1 | 0.238 |
| Child’s sex | 1.739 | 1 | 0.187 |
| Child’s birth weight | 3.105 | 1 | 0.078 |
| Father’s age | 0.289 | 1 | 0.591 |
| Father’s education (three classes)^b^ | 2.466 | 2 | 0.291 |
| Vocational school/Polytechnic | 1.179 | 1 | 0.278 |
| University | 2.383 | 1 | 0.123 |
| Previous children (three classes)^c^ | 4.720 | 2 | 0.094 |
| One | 1.188 | 1 | 0.276 |
| Two or more | 2.282 | 1 | 0.131 |

^a^ Test statistic based on the change in model log-likelihood associated with the effect. Threshold for entry to the model p < 0.05.

^b^ None/Some vocational training as the reference category

^c^ No children as the reference category
